# High-risk HPV prevalence and vaccination coverage among Indigenous women in the Colombian Amazon: Implications for cervical cancer prevention. Cross-sectional study

**DOI:** 10.1371/journal.pone.0297579

**Published:** 2024-02-05

**Authors:** María Inés Sarmiento-Medina, Miryam Puerto de Amaya, Licet Villamizar-Gómez, Andrea Carolina González-Coba, Laura Guzmán-Barajas

**Affiliations:** 1 Vicerrectoría de Investigaciones, Fundación Universitaria de Ciencias de la Salud, Bogotá D.C, Colombia; 2 Facultad de Tecnologías en Salud, Fundación Universitaria de Ciencias de la Salud, Bogotá D.C, Colombia; 3 Departamento de Patología, Fundación Universitaria de Ciencias de la Salud, Bogotá D.C, Colombia; Ruđer Bošković Institute, CROATIA

## Abstract

Cervical cancer, primarily caused by Human Papillomavirus (HPV) transmission through sexual contact, necessitates comprehensive strategies to combat its impact on women’s health. Yet, certain underserved populations, such as low socioeconomic and ethnic minority groups, encounter barriers in accessing timely interventions and early diagnosis. This cross-sectional study was conducted with the aim of assessing HPV prevalence, genotype distribution, and co-infections among 280 adult women residing in a Colombian Indigenous Reserve within the Amazon region. The research adhered to a community-centric approach that respected cultural norms, native languages, and Indigenous authorities’ permission. The study revealed an overall HPV infection prevalence of 31.1% (n = 87, 95% CI 25.7–36.8), with 22.5% (n = 63, 95% CI 17.7–27.8) of women infected by at least one high-risk HPV genotype and 15.0% (n = 42, 95% CI 11–19.7) infected by at least one LR genotype. These results align with the findings of other Colombian studies. Notable high-frequency genotypes included *16*, *52*, *66*, *56*, and *68*, with the most common combinations being [*66*–*52*] and [*66*–*58*]. The study also assessed the prevalence of HPV vaccination, revealing a rate of 22.9%, lower than the national average. In vaccinated women, the prevalence of genotypes *16* and *18* was significantly reduced, as anticipated. Importantly, it was observed that 57.1% of all high-risk HPV infections could have been prevented with the use of the nonavalent vaccine. These findings underscore the critical need to enhance adherence to early cervical cancer detection and monitor positive cases to evaluate high-risk HPV persistence. Efforts should be directed toward continuing vaccination coverage against high-risk HPV *16* and *18* with the quadrivalent vaccine, while also striving to make the nonavalent vaccine accessible for inclusion in large-scale public health programs. Additionally, the study did not identify a specific pattern of co-infection. The study emphasizes the significance of adopting a locally tailored epidemiological approach to guide and promote cervical cancer prevention efforts in Indigenous communities.

## Introduction

Cervical Cancer (CC) represents a significant global burden within the realm of gynecological pathologies, standing as the fourth most prevalent cancer among women [1]. Over time, comprehensive strategies have been devised to mitigate the impact of this disease, focusing on alleviating its morbidity, facilitating early detection, and reducing mortality rates [2]. The well-established etiological agent is the Human Papillomavirus (HPV) [3], a sexually transmitted pathogen. CC can be prevented through targeted interventions such as vaccination, HPV testing, and cervical-vaginal cytology. However, certain low socioeconomic and ethnic minority groups find barriers when it comes to accessing these vital measures for intervention and early diagnosis [4].

Studies are being carried out on the influence of co-infection on the risk of cervical lesions, as well as on the tendency of HPV genotypes to cluster in a certain way. Their results show weak or non-existent relationships [5–8]. Reports in Colombia show significant prevalence of HPV co-infection such as 85% [9], 37.1% [10], and 24.9% [11].

Regarding immunization, in Colombia, the public health programs included the vaccine against HPV infection for girls over 9 years of age as a primary prevention measure against CC since 2012 [12]. Currently, the quadrivalent vaccine (4v) is applied with a coverage of 34% for the first dose and 11% for the second dose. These coverages are considered low, and cultural reasons are the main causes of this situation [13]. They are expected to be lower in Indigenous population [4].

This study shows the prevalence and distribution of the different types of HPV in women from a Colombian Indigenous group. The frequency of risk factors, the type of single or multiple infection, the cervical cytology result in high-risk HPV (HR-HPV) positive cases and HPV vaccination status are analyzed. Thus, it provides knowledge about HPV infection and offers a baseline for strengthening local vaccination and CC screening programs.

## Methods

A descriptive cross-sectional study was done to analyze the frequency of HPV infection, type of HPV, either high or low risk (LR), and the frequency of co-infection in women who belong to the Paujil Indigenous Reserve in the department of Guainía, Colombia.

### Population and sample

Convenience sampling was done since the Indigenous authorities on the reserve have no records of their population. An estimated total of 4,000 people was calculated of which 40% were assumed to be adults and, of these, half were assumed to be women thus leading to consideration of 800 adult women.

All adult women living on the reserve were invited to the health center for CC prevention screening. The cervical samples were taken at the center in three sessions over a six-day period in July, October, and December 2022. Before starting, informative sessions were held in the five native languages most frequently spoken in this reserve with the support of trained Indigenous leaders and the use of flyers, posters, and house-to house visits.

### Inclusion and exclusion criteria

To participate in the study, 305 women attending cytology sample collection at the health center were evaluated regarding inclusion and exclusion criteria. The inclusion criteria were women: a) belogning to the Paujil Indigenous Reserve; b) having started sexual relations at least three years before screening (regardless of age); c) not pregnant; d) not menstruating at the time of the screening; e) younger than 75 years; f) accepting collecting sample procedure. The exclusion criteria were: a) identifying oneself as not belonging to an Indigenous group; b) patients who refused to participate in research.

Among this group, 24 women not identifying with any Indigenous group were excluded of the study because they complied with one of the exclusion criterion. However, for ethical reasons, the screening test was done. One Indigenous woman did not accept collecting cervical sample, so she was not included. Eventually, the study sample was 280 Indigenous women.

### Clinical specimen collection

Liquid-based cervical samples from endocervix and exocervix were taken using Roberts^®^ cytobrushes and preserved at room temperature in 20 ml of preservative solution (PreservCyt^®^) until processing 96 hours after. HPV-DNA was screened for 19 HR and 9 LR genotypes through the use of the Anyplex II^®^ Real Time Polymerase Chain Reaction (TR-PCR) HPV 28 by SEEGENE^®^ detection technique. The high oncogenic risk viral genotypes (HR-HPV) were: *16*, *18*, *26*, *31*, *33*, *35*, *39*, *45*, *51*, *52*, *53*, *56*, *58*, *59*, *66*, *68*, *69*, *73*, *82*. The LR-HPV genotypes were: *6*, *11*, *40*, *42*, *43*, *44*, *54*, *61 and 70*.

### Sociodemographic features and CC risk factors

Sociodemographic features such as age, socioeconomic level, ethnicity, and educational level were considered. The socioeconomic level classification was based on the indicator used by the National Planning Department of Colombia, which indirectly measures the poverty or wealth level. It takes into account the geographic location, the physical characteristics, and the urban context of settlements. It defines six categories or strata with strata 1 reflecting households with the lowest spending capacity and strata 6 reflecting those with the highest capacity. For this study, strata 1 and 2 were classified as very low and low, respectively, strata 3 and 4 as medium, and strata 5 and 6 as high [14].

Because the Paujil Reserve is home to people who belong to several Indigenous groups, as well as some people who feel they do not identify with any group, the women were asked about their race based on the group to which they felt they belonged. The Indigenous groups found in the sample were Puinave, Sikuani, Piapoco, Cubeo, Curripaco, Tucano, and Yeral.

Known CC risk factors such as age at first sexual intercourse, lifetime sexual partners and HPV vaccine were explored. All of the patients were asked about them directly, in Spanish and with a translator woman in case it was necessary. The number of sexual partners was grouped as follows: 1, 2–3, 4–5, and 6 or more.

The type of HPV infection was classified as single when just one genotype was detected in the sample and multiple when there were two or more different genotypes.

The HPV-positive samples underwent liquid-based cytology. The analyses were done in a laboratory in Bogota, the capital of the country. Abnormal cytology was defined according to the Bethesda classification, including the following categories: Atypical squamous cells of undetermined significance (ASC-US); Atypical squamous cells that cannot exclude high-grade squamous intraepithelial lesions (ASC-H); low-grade squamous intraepithelial lesion (LSIL); high-grade squamous intraepithelial lesions (HSIL), and atypical glandular cells (AGC).

### Ethical aspects

The study followed the ethical guidelines established by the Declaration of Helsinki (2013) and was reviewed and approved by the Ethics Committee of Hospital San Jose de Bogota (Certificate N° 9/2017) as well as by the Indigenous Reserve authorities.

All the women received information in their native language and in Spanish before signing the informed consent document. Minors (<18 years of age) who already had a family signed the consent form themselves, as they are considered adults in Indigenous communities and can make their own decisions.

During the data analyses, all information was handled anonymously through the use of codes including one for ethnicity in order to avoid possible discrimination. The DNA-HPV and liquid-based cytology test results were only labeled when they were given to each patient and when they were sent to the management staff at the local hospital for follow-up and individualized care. The results were given to the patients with a suggestion of next control and with an explanation to the patients by the health staff.

### Statistical analyses

A descriptive analysis of demographic characteristics and risk factors was done. These were treated as categorical variables. Quantitative variables were reported using medians and ranges. The Confidence Interval (CI) for HPV prevalence was obtained from Stata version 13.0^®^ program at 95% level, however as the sample was not representative, it was not possible to make any inference to the general population. The strength in the association of the independent variables with respect to HPV infection was measured using Odds Ratio with 95% CI. The CI for the differences of prevalence by age groups was calculated at 95% level. Either the Chi-Square Test or the Fisher’s Exact Test were used to assess the difference between the proportions. Missing data were accounted for in the denominator of each proportion. The statistical analysis was done using the Stata version 13.0^®^ program and Microsoft Excel^®^ graphs.

## Results

### Characteristic of the study population

The study included 280 women from seven different Indigenous groups who met all inclusion criteria. The median age was 39 years (range: 17–74). Of the total, 81.9% (n = 223) had primary or secondary level of education; 75% (n = 204) were married or lived in consensual union; and 93.7% (n = 255) had a very low or low socioeconomic status. The prevalence of HPV infection in the study population was found to be 31.1% (95% CI 25.7–36.8 n = 87/280). (Table 1).

**Table 1:** Participant characteristics and risk factors associated with HPV infection in an Indigenous reserve in Colombia.

Description	Total n (%)	HPV Positive 87/280 (31.0%)	HPV Negative 193/280 (68.9%)	OR (95% CI)	P value
*Age (years)*, *median (range)*	39 (17–74)	31 (17–69)	41 (17–74)		
< 30	81 (28.9)	37 (42.5)	44 (22.8)	0.39 (0.22–0.69)	**0,001** *
≥ 30	199 (71.1)	50 (57.5)	149 (77.2)	Reference	
*Ethnic Group* ***					
1	124 (44.2)	37 (42.5)	87 (45)	Reference	0.12**
2	39 (13.9)	12 (13.8)	27 (14)	1.05 (0.48–2.28)	
3	36 (12.9)	8 (9.2)	28 (14.5)	1.48 (0.62–3.57)	
4	31 (11.07)	9 (10.3)	22 (11.4)	1.03 (0.44–2.47)	
5	21 (7.5)	6 (6.9)	15 (7.8)	1.06 (0.38–2.95)	
6	19 (6.8)	12 (13.8)	7 (3.7)	**0.25 (0.09–0.68)**	
7	10 (3.6)	3 (3.5)	7 (3.6)	0.99 (0.24–4.05)	
*Education level*, *n = 272*					
Primary or secondary	223 (81.9)	68 (80.0)	155 (82.9)	0.82 (0.42–1.59)	0.56 *
University	49 (18.1)	17 (20.0)	32 (17.1)	Reference	
*Marital status*, *n = 272*					
Married or cohabiting	204 (75)	56 (67.5)	148 (78.3)	Reference	0.16 *
Single	42 (15.4)	17 (20.5)	25 (13.2)	1.79 (0.89–3.59)	
Divorced or widowed	26 (9.6)	10 (12)	16 (8.5)	1.65 (0.7–3.87)	
*Socioeconomic status*, *n = 272*					
Very low / low	255 (93.7)	79 (91.9)	176 (94.6)	Reference	0.38 *
Medium	17 (6.3)	7 (8.1)	10 (5.4)	1.55 (0.57–4.26)	
*Age at first sexual intercourse (years)*, *n = 265*					
*≤ 14*	64 (24.2)	20 (24.4)	44 (24.1)	1.01 (0.55–1.87)	0.95 *
*> 14*	201 (75.8)	62 (75.6)	139 (75.9)	Reference	
*Lifetime sexual partners*, *n = 202*					
*1*	75 (37.1)	14 (25)	61 (41.8)	Reference	0.15 *
*2–3*	70 (34.7)	23 (41.1)	47 (32.2)	2.13 (0.97–4.64)	
*4–5*	27 (13.3)	10 (17.8)	17 (11.6)	2.56 (0.94–6.94)	
*≥ 6*	30 (14.9)	9 (16.1)	21 (14.4)	1.86 (0.69–5)	
*Previous HPV vaccine*, *n = 279*					
Yes	64 (22.9)	24 (27.9)	40 (20.7)	Reference	0.18 *
No	215 (77.1)	62 (72.1)	153 (79.3)	0.67 (0.37–1.21)	

*chi-square test;

** Fisher’s exact test.

*** It has been coded to protect confidentiality.

The age of first sexual intercourse at 14 years or younger was registered for 24.2% (n = 64); 62.9% reported having more than one sexual partner in their lifetime and 77.1% (n = 215/279) had not been previously vaccinated against HPV.

Being younger than 30 years was a protector factor against HPV infection (OR: 0.39; 95% CI: 0.22–0.69; p value = 0.001). The difference of proportions of HPV infection within these two age groups was 0.15 (95%CI: 0,022–0.2778) p value = 0.0113. No statistically significant difference was found for any of the following conditions: educational level, marital status, socioeconomic level, ethnic group, age at first sexual intercourse, number of sexual partners, and previous HPV vaccination (Table 1).

### Distribution and characteristics of HPV infection

Among all individuals, 22.5% (95% CI: 17.7–27.8 n = 63/280) tested positive for at least one HR-HPV genotype, while 15.0% (95% CI: 11–19.7 n = 42/280) were positive for at least one LR-HPV genotype. Nearly half of the HPV infections (42.5%) were observed among women belonging to the number 1 Indigenous group. This proportion remained consistent for HR-HPV infections, accounting for 46.0% of the cases. The comparations through Odds Ratio was not statistically significant except when it was compared Group 1 with Group 6 (Table 1) where Group 6 showed less probability of having HPV infection than Group 1.

Regarding the history of vaccination against HPV, 22.9% (64/279) of women reported being vaccinated. Among these vaccinated individuals, 37.5% (24/64) showed HPV infection.

**Fig 1:**
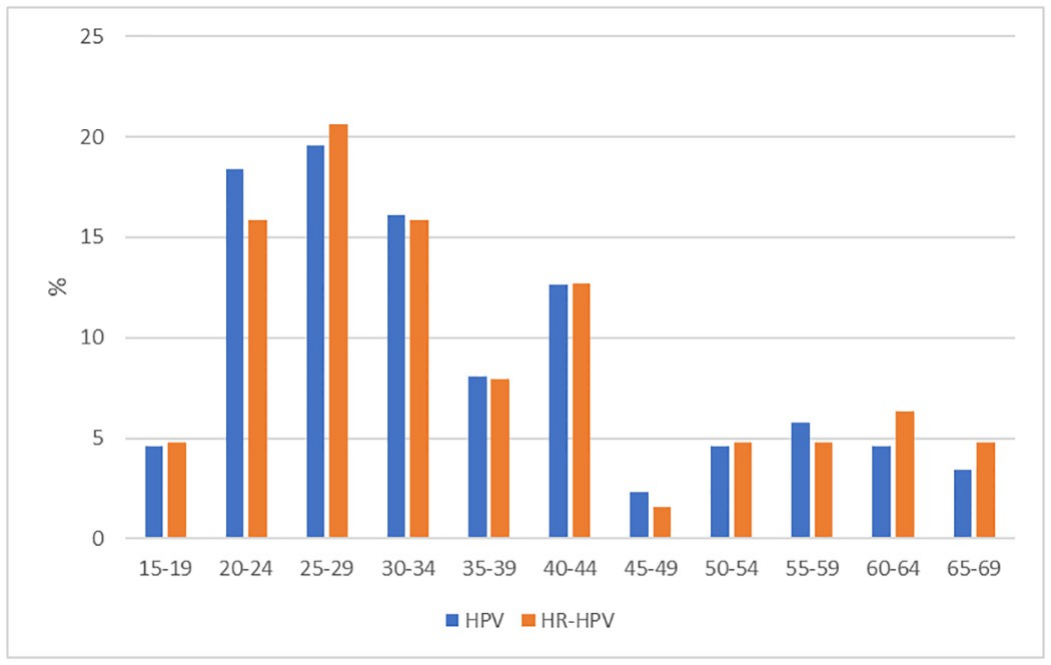
Distribution of Global and HR-HPV infections by age in an Indigenous reserve in Colombia.

In terms of age distribution, HPV infection exhibited a bimodal pattern, with a higher peak observed among individuals aged 20 to 35 years and a smaller peak among individuals aged 40 to 44 years. The highest prevalence of HR-HPV was observed in the group of 25–29 years. (Fig 1).

The group of 87 patients with HPV infection underwent liquid-based cytology with normal results in 80.5% (n = 70). ASCUS occurred in 17.2% (n = 15) of cytological abnormalities. No cancer was detected. Table 2 shows these results broken down by HPV vaccination status. When comparing normal results in vaccinated women versus those unvaccinated, no significant difference was found (Fisher’s exact test p value = 0.54).

**Table 2:** Results of liquid-based cytology of cervical samples from HPV positive women from an Indigenous reserve in Colombia.

	HPV Positive n = 87 (100%)	Vaccinated n = 24n (100%)	Not vaccinated n = 62 (100%)
*Liquid-based cytology*			
Normal	70 (80.5)*	18 (75)	51 (82.3)
ASC-US	15 (17.2)	5 (20.8)	10 (16.1)
LSIL	1 (1.1)	1 (4.1)	0
HSIL	0	0	0
Not discarded HSIL	1 (1.1)	0	(1.6)

*One woman with no information of vaccination status.

Among the individuals infected with HPV, 59.8% (n = 52/87) showed a single genotype while 40.2% (n = 35/87) were infected with 2, 3, 4, or 7 genotypes. There was no association with vaccine status (chi-square test p value = 0.90) (Table 3).

**Table 3:** Single or multiple infection in women HPV positive from an Indigenous reserve in Colombia.

*Number of simultaneous genotypes*	HPV Positive n = 87 (100%)	Vaccinated n = 24n (100%)	Not vaccinated n = 62n (100%)
1	52 (59.8)	14 (58.3)	37 (59.7)
2	20 (23.0)	6 (25.0)	14 (22.6)
3	10 (11.4)	4 (16.6)	6 (9.7)
4	4 (4.7)	0	4 (6.5)
7	1 (1.1)	0	1 (1.6)

Among all HR-HPV infections, 19.0% (16/84) was produced by HPV *16* or *18* and 57.1% (n = 48/84) by one or more among the seven HR-HPV genotypes that nonavalent vaccine (9v) prevents (Table 4).

**Table 4:** Distribution of HR-HPV genotypes found in 63 women from an Indigenous reserve in Colombia.

HR-HPV Genotype	Frequency of positive result* n (%)	Vaccinated n (%)	Not vaccinated n (%)
*16*	12 (14.3)	2 (2.4)	10 (11.9)
*18*	4 (4.8)	2 (2.4)	2 (2.4)
*31*	7 (8.3)	1 (1.2)	6 (7.1)
*33*	4 (4.8)	2 (2.4)	2 (2.4)
*45*	4 (4.8)	1 (1.2)	3 (3.6)
*52*	9 (10.7)	2 (2.4)	7 (8.3)
*58*	8 (19.5)	1 (1.2)	7 (8.3)
Total HR included in 9v vaccine	48 (57.1)	10 (11.9) **	37 (44.0)
Other HR- HPV	36 (42.9)	8 (8.3)	28 (33.3)
Total HR-HPV positive results	84 (100)	18 (20.2)	65 (77.4)

*One woman may have more than one infection.

**No data of vaccination status in one woman HR-HPV (+).

**Fig 2:**
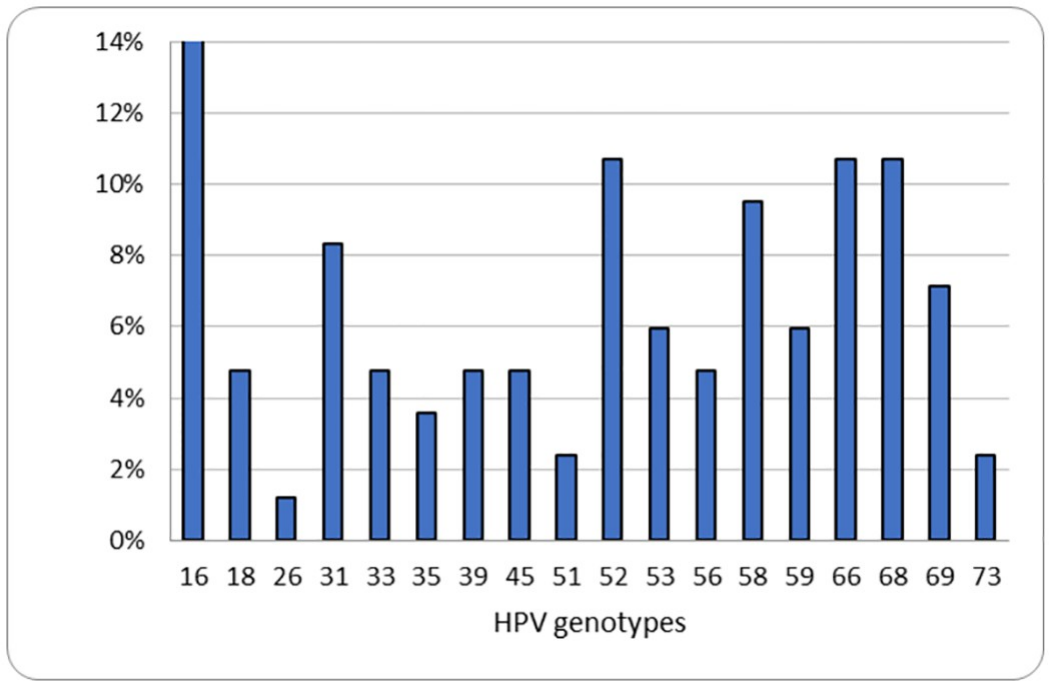
HR-HPV genotypes in 63 women from an Indigenous reserve in Colombia.

The most frequent genotype was *16* at 14.3% (n = 12) followed by *52*, *66*, and *68* at an equal frequency of 10.7% (n = 9) (Fig 2).

**Fig 3:**
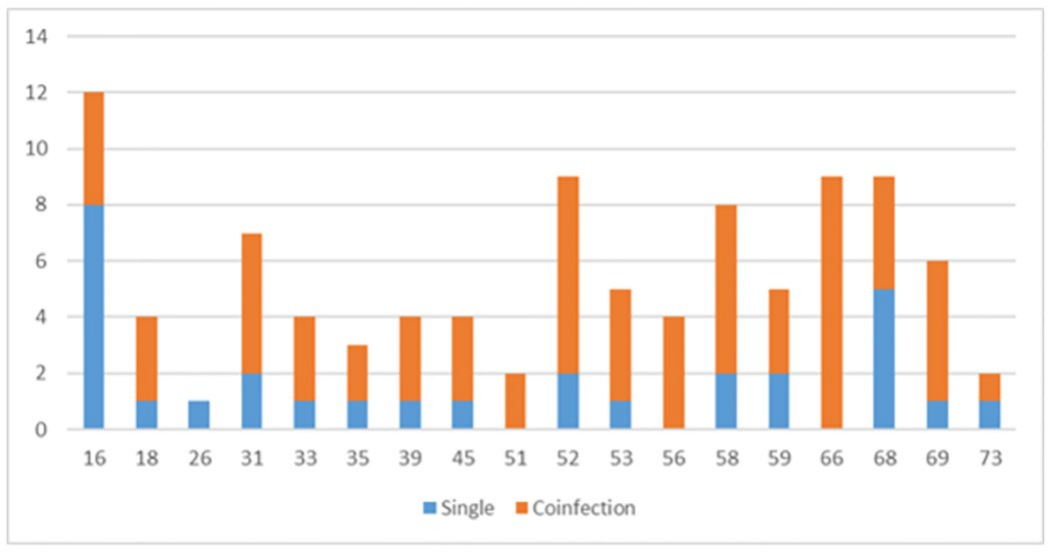
Distribution of HR-HPV based on single or co-infection presentation in women in an Indigenous reserve in Colombia.

Among the HR genotypes, the *16* was notable with a high frequency of single infection while types *18*, *31*, *52*, *53*, *58*, and *69* showed a high frequency of multiple infections. Genotypes *51*, *56*, and *66* only appeared as co-infection (Fig 3).

No combination of HPV genotypes stood out in terms of frequency (Table 5).

**Table 5:** HR-HPV genotypes combination in women from an Indigenous reserve in Colombia.

Type of HR-HPV co-infection (n = 35)	n (%)
*66–52*	3 (8.6)
*66–58*	3 (8.6)
*56–51*	2 (5.7)
*58–52*	2 (5.7)
*59–53*	2 (5.7)
*56–51*	2 (5.7)
*66–58*	2 (5.7)
*Other*	19 (54.3)

Regarding the distribution of LR-HPV (n = 42), there was no infection for genotypes *6* or *11* in vaccinated women and it was really low in not vaccinated group (Table 6).

**Table 6:** Distribution of LR-HPV found in women from an Indigenous reserve in Colombia.

LR-HPV Genotype	LR-HPV Positive n = 42 (100%)	Vaccinated n = 14 (100%)	Not vaccinated n = 27 100%)
*6*	2 (4.8)	0	2 (7.4)
*11*	1 (2.4)	0	1 (3.7)
*Other LR-HPV no included in HPV vaccines*	40 (95.2)	14 (100)	25 (92.6)

**Fig 4:**
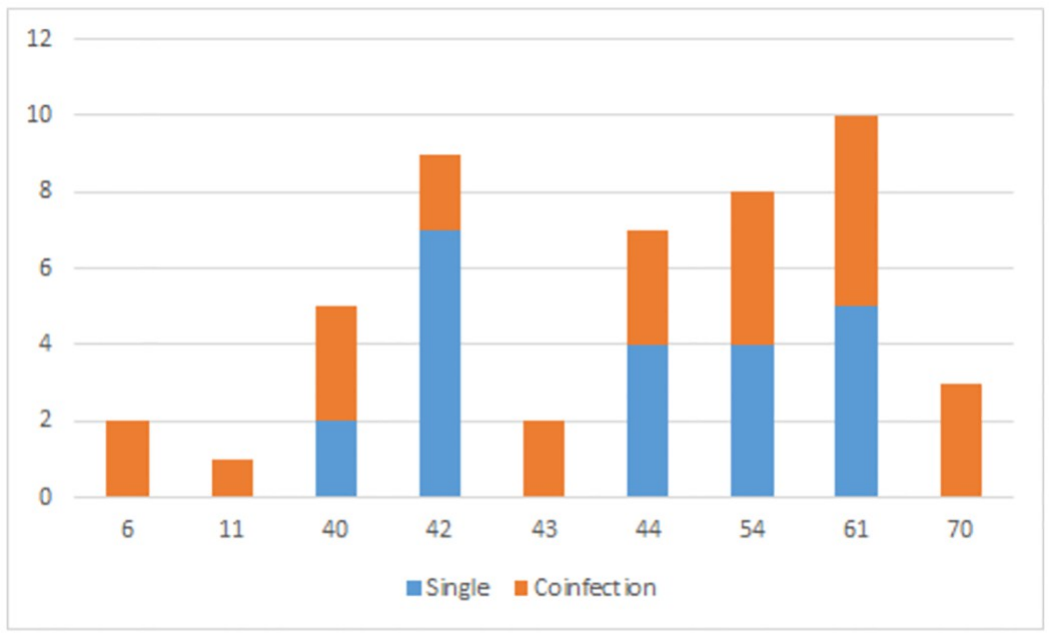
Distribution of LR-HPV based on single or multiple infection in women from an Indigenous reserve in Colombia.

For the LR-HPV, genotypes *40*, *42*, *44*, *54*, and *61* occurred as co-infection, while genotypes *6*, *11*, *43*, and *70* occurred as single infection (Fig 4).

## Discussion

This study describes the epidemiological characteristics of HPV infection of a population group with particular race and cultural attributes that deserve a special approach. Thus, it contributes to decrease the social gap related to Indigenous communities around the world [15,16]. Considering the fact that local health services do not routinely search for viral DNA and favor cytology as a screening mechanism for CC, this study provides more accurate information with which to work on the prevention and early detection in the women of the Paujil Reserve. Knowing the distribution and characteristics of HPV infection in each population allows for better targeting programs for vaccination and prevention of neoplastic lesions in CC.

The epidemiological distribution of HPV infection was analyzed in a population group of 280 women from seven Indigenous ethnic groups in the Colombian Amazon living on an Indigenous Reserve. An overall prevalence of HPV infection of 31.1% was found, which is within a wide range of prevalence reported by other studies in Colombia: Torrado et al. [17] reported 10% in the lower income strata of the mestizo population in a Colombian city; Angarita & Forero [9] found 47.4% in one group of Indigenous population; Camargo [11] found 49.6% in five Colombian cities, and Puerto et al. [10] found 60.3% in women aged 18 to 25 years in Bogota. In other Latin American countries, Carrión et al. [18] reported a prevalence of 34% in an Ecuadorian Indigenous population, Martorell et al reported 35.4% in a Peruvian Indigenous population [19] and Bobadilla et al. [20] in Paraguay reported a prevalence of 54.8%. This variation is found throughout the Latin American region as described by Moya and Rojas [21], who reviewed 39 publications in which HPV infection prevalence ranged from 6.7% to 82.2%. This extensive range of prevalence suggests the idea that the HPV infection is not easy to compare. It depends of the settings and the time when this has been measured, the groups of age that have been included, the race and the way this is determined, and even the capability of the test to detect more or fewer genotypes. Comparing the prevalence of infection in the same population group over time might offer more useful information to support local preventive measures.

In this study, HPV infection exhibited a bimodal pattern that was age-related. High prevalence was observed in younger women, which decreased in older women and then showed a slight increase again from the age of 40. This trend is similar to the one reported by Dalstein et al. [22] and different from that of Liao et al. [23] who found a very distinct U-shaped pattern.

The 22.5% prevalence of HR-HPV found in this study is considered high when compared to some reports in Colombia such as Torrado’s et al [17] that found 3.9% and Perez’s et al [24] with 11.5%. However, it is lower when comparing with other Colombian studies, for example, the one of Puerto et al. [10] that reported 42.2%. In other Latin American countries such as Paraguay, Costa Rica, Ecuador or Peru it has been found 42.3% [20]; 33.8% [5]; 34% (Indigenous population) [18] and 35.4% (also Indigenous population) [19] respectively. In contrast, the multicenter Latin American ESTAMPA [25] study of 31,438 women found a much lower prevalence of 14%.

When the prevalence of HR-HPV is compared, it must be borne in mind that they may vary depending on the ability of the test to detect a given number of genotypes. The greater the ability to detect diverse genotypes, the more likely it is that a higher prevalence will be found. In this study, a test was used to detect 19 HR-HPV and 14 LR-HPV genotypes. Other studies used tests that detect only two specific HR-HPV (*16* and *18*) and 14-grouped HR-HPV [24] and *17* HR-HPV and *10* LR-HPV [23].

Regarding the variety of genotypes, our findings coincide with the literature in that the most frequent is type *16*. However, its proportion within the total HR-HPV of 19% was lower than the reports of other Colombian studies: 46.2% [4], 36.1% [11], and 33.9% [10]. Genotype *18* was among the least frequent with only 6.3%. This low frequency coincides with other Colombian studies in which it does not appear among the most prevalent [10,11,24].

The preponderance of type *16* is maintained, even though genotypes other than *16* and *18* hold significant positions in the frequency distribution in both the population in this study and in other Colombian and Latin American populations. In the communities of the Paujil Reserve, genotype *16* was the number one genotype, followed by *52*, *66* and *68*, all in second place. Angarita & Forero [9] found genotype *16* to be the number one followed by genotypes *31* and *39*; Cómbita et al. [26] also described genotype *16* as number one, followed by *52*, *58* and *51* in an unvaccinated population of the city of Bogota. Puerto et al. [10] reported genotype *16* as number one, followed by *52* and *51*. Paraguayan study of Bobadilla et al. [20] reported that the most frequent genotype was *58*, followed by *16*, *51*, and *66*. These data might suggest that infections with the various HPV genotypes do not have a prominent pattern (except for the preponderance of HPV *16*) and tend to have variations between regions.

In this study, differences between the genotypes presented in vaccinated and unvaccinated women were observed. HR *16* and *18* genotypes have arguably disappeared in vaccinated women. The few cases found may be attributed to recall bias in relation to the history of vaccination. It can be stated that 19.0% of HR-HPV infection (those with *16 or 18* genotypes) could have been prevented with 4v or 9v vaccine and 57.1% (those with *16*, *18*, *31*, *33*, *45*, *52* or *58* genotypes) could have been prevented with 9v vaccine. This vaccine, unfortunately, is not accessible for mass vaccination programs in Colombia because of its high cost (approximately US$ 115 per dose). Therefore, prevention of oncogenic viruses is carried out only for the *16 and 18* genotypes through the 4v vaccine which is free of charge for the general population.

In this regard, several researchers have drawn attention to the importance of genotypes other than *16 or 18* with respect to cervical lesions. Kabaca et al. [27] state that a quarter of CC cases are related to HR-HPV other than *16 or 18*. In their study of colposcopies of 752 patients over 30 years of age in Turkey with negative cytology and HR-HPV infection other than *16 or 18*, they found pre-invasive lesions, especially in women with genotypes *31*, *33* and *39* so they suggest that patients with these genotypes should be evaluated by colposcopy even if they have negative cytology. Bai et al. [28] reached similar conclusions based on their study of 3,091 patients with normal cytology and HPV infection in China. They found that infections with HPV genotypes *31*, *33*, *35*, *52*, and *58* are significant risk factors for cervical lesions and that patients with multiple infections with HPV *31*, *33*, and *52* should be included in colposcopy screening since the benefit of prevention may outweigh the disadvantages of increased colposcopy in medical services.

Associations between cervical lesions and infection with HR-HPV other than genotypes *16 or 18* are also notable in the study by Gaete et al [29] on 213 samples of cervical pathology with a diagnosis of dysplasia or cancer in which *31* is found to be the number one genotype at 47%, followed by genotypes *33*, *16*, *44*, and *28*.

With respect to co-infection, no pattern was found in this study. The prevalence reported by different researchers is variable in Colombian studies such as the ones by Angarita & Forero [9] who reported 85.6%, Puerto et al. [10] 37.1% and Camargo [11] 29.4%. Regarding other countries, co-infection prevalence of 18.2% has been found in Costa Rica [5], 61% in Denmark in women under 30 years of age and 39% in women over 30 years of age [30], and 18.1% in a group of four Nordic countries in Europe [31]. In our study the prevalence of co-infection in women with HPV was 40.2% (n = 35/87).

It is not possible to establish if the differences found with respect to other population groups in Colombia or in other countries in terms of HPV prevalence and genotypes are due to the ethnic or cultural characteristics of the Indigenous groups. Additionally, with the information obtained from this study, it cannot be stated that one of the seven Indigenous groups included had a greater probability of HPV infection. Studies aimed at exploring this hypothesis are required.

Some researchers suggest concentrating screening on the genotypes most frequently associated with CC, like Sundström and Dillner [32], who found that 85.3% of the cases of invasive cancer corresponded to genotypes *16*, *18*, *31*, 33, *45*, *52*. In consequence, they consider inefficient to test for 14 types of viruses of which there are eight with very little association with CC. Nygard et al. [31] agree with the idea of reducing the number of genotypes that are explored in screening. They have stated that, although tests including 14 oncogenic viruses can detect low-grade lesions in the post-vaccination era, the best screening performance in terms of detecting high-grade lesions will be obtained by focusing on the HPV genotypes covered by the 9v vaccine.

The proportion of women vaccinated against HPV with at least one dose in this Indigenous community (22.9%) is lower than the one of the whole country reported by Castro at 34% [13]. The results show that HPV vaccination has protected vaccinated women from acquiring infection against the genotypes most closely related to CC, therefore, vaccination coverage should be further expanded. Considering the fact that 57.1% of the HR-HPV infections found in this group of Indigenous women are caused by genotypes that are preventable with the 9v vaccine, it is essential to move forward to acquire it at affordable costs for the country in order to include it in mass programs.

It is also important to follow patients with HR-HPV to detect persistence of infection because of its relevance in the development of precancerous lesions [22]. The Colombian Ministry of Health guidelines [33] recommend an immediate colposcopy for women over 30 years of age after two consecutive positive tests with an interval of 18 months if they have normal cytology. For women with abnormal cytology and infected with HR-HPV, immediate colposcopy is recommended.

Based on the experience of this study, one might consider that fluctuating or highly mobile populations, who carry risk factors and have low adherence to screening—such as women from some Indigenous communities- could benefit from an immediate search for cervical lesions when they test positive for the seven most frequent HR-HPV infection in any of the screenings. As their lifestyles and especially their mobility make controls very sporadic, it is very difficult to know if the infection would become persistent or not. Therefore, it is essential to continue improving CC screening strategies in these population groups [34].

One of the limitations of this study is the size and representativeness of the sample since it was not possible to obtain a census to ensure that it was representative of the population. Another limitation is that 20.0% of the data on the number of sexual partners was missing, and this may have contributed to the lack of association between this factor and HPV infection. It should be noted that this study was carried out with Indigenous communities with the difficulties that this entails such as language, customs, and lack of trust in health professionals. These aspects were addressed by the cultural adaptation of screening activities. The study provides local health authorities with information on the amount of the adult female population with HPV infection. This can serve as a baseline for evaluating the impact of vaccination as stated by Garland et al. [35], and as a stimulus for improving education and vaccination coverage. Moreover, this study may be useful for maintaining adherence to screening programs by designing public heath interventions that take ethnic diversity into account [17]. In addition to contributing to the knowledge of HPV infection and coinfection in women, it helps to reduce the health services gap related to Indigenous peoples in the world [34] and specifically in this Amazon reserve.

This protocol was developed following the Standard Protocol Items: STROBE Iniciative (Strengthening the Reporting of Observational Studies in Epidemiology) checklist (see S1 Checklist) [36].

## Supporting information

S1 Checklist STROBE statement—Checklist of items that should be included in reports of observational studies. (PDF)
