# Supplementary material for: High-risk HPV prevalence and vaccination coverage among Indigenous women in the Colombian Amazon: Implications for cervical cancer prevention. Cross-sectional study
Source: PLoS One. 2024 Feb 5;19(2):e0297579. doi: 10.1371/journal.pone.0297579 (PMC10843138; doi:10.1371/journal.pone.0297579)
Supplement: S1 Checklist — (PDF) [file pone.0297579.s001.pdf]

STROBE Statement—checklist of items that should be included in reports of observational studies

High-Risk HPV prevalence and vaccination coverage among Indigenous women in the Colombian Amazon: implications for cervical cancer prevention. Cross-sectional study.

	Item No.	Recommendation	Page No.	Relevant text from manuscript
**Title and abstract**	1	(*a*) Indicate the study's design with a commonly used term in the title or the abstract	1	Cross sectional study
		(*b*) Provide in the abstract an informative and balanced summary of what was done and what was found	2	Yes
**Introduction**				
Background/rationale	2	Explain the scientific background and rationale for the investigation being reported	4,5	Cervical cancer necessitates comprehensive strategies to combat its impact on women's health. Certain underserved populations, such as low socioeconomic and ethnic minority groups, encounter barriers in accessing timely interventions and early diagnosis. Vaccination coverages are low in Colombia and they are expècted to be low in Indígenous communities. This study shows the prevalence and distribution of the different types of HPV in women from a Colombian Indigenous group. The frequency of risk factors, the type of single or multiple infection, the cervical cytology result in high-risk HPV (HR-HPV) positive cases and HPV

				vaccination status are analyzed
**Objectives**	3	State specific objectives, including any prespecified hypotheses	5	-To analyze the frequency of HPV infection, type of HPV, either high or low risk (LR), the frequency of co-infection and vaccination status in women who belong to the Paujil Indigenous Reserve in the Department of Guainía, Colombia<br>Cross sectional study

Methods

Study design	4	Present key elements of study design early in the paper	1<br>1<br><br><br>4,5<br><br><br><br><br><br><br><br>6<br><br><br>8	- Title: Cross sectional study<br>- Title: prevalence of HPV<br>-<br>- Introduction:<br>- This study shows the prevalence and distribution of the different types of HPV in women from a Colombian Indigenous group.<br>- Participatory strategy was used considering the cultural characteristics and native languages<br>- Samples of cervical cells were taken to be analyzed for explore DNA from HPV<br>-Cytology was done to the positive samples.
Setting	5	Describe the setting, locations, and relevant dates, including periods of recruitment, exposure, follow-up, and data collection	5<br><br><br>6<br><br>6<br><br><br>5<br><br>6	-Reserve of Paujil. Departmentof Guanía, Colombian Amazonic Region<br>- Recruitment during second half of 2022<br>-Women liders make aneducative bilingual campaign in communities<br>Women assisted voluntarily to a screening call.<br>Prospective data collection

Participants	6	(*a*) *Cohort study*—Give the eligibility criteria, and the sources and methods of selection of participants. Describe methods of follow-up<br>*Case-control study*—Give the eligibility criteria, and the sources and methods of case ascertainment and control selection. Give the rationale for the choice of cases and controls—Give the eligibility criteria, and the sources and methods of selection of participants	6<br>5<br>6	*Cross-sectional study*<br>Sources: Women living in the Indigenous Reserve of Paujil<br>Methods of selection: 305 women who went to the health center asking for screening test.<br>Eligibility criteria:<br>-Women between 17 – 75 years<br>-Identified as belonging to an ethnic group<br>-Have had sexual intercourse for at least 3 years before the screening.<br>-Not menstruating.<br>-Not pregnant<br>-Previous informed consent
		(*b*) *Cohort study*—For matched studies, give matching criteria and number of exposed andunexposed<br>*Case-control study*—For matched studies, give matching criteria and the number of controls per case		
Variables	7	*Clearly define all outcomes, exposures, predictors, potential confounders, and effect modifiers. Give diagnostic criteria, if applicable*	12<br>8	-Outcomes:<br>- Results of HPV DNA tested for 19 oncogenic high-risk (*16*, *18*, *26*, *31*, *33*, *35*, *39*, *45*, *51*, *52*, *53*, *56*, *58*, *59*, *66*, *68*, *69*, *73* and *82)* and 9 low-risk genotypes (*6*, *11*, *40*, *42*, *43*, *44*, *54*, *61*, and *70)* using the Real-Time Polymerase Chain Reaction<br>- Single HPV infection was defined as only one genotype if virus in one sample. Co- infection was

				defined as two or more different genotypes in one sample.
			8	- Results of cytology in women HPV-HR positive: defined as Bethesda classification
				· Exposures
			7	Sociodemographic data: socioeconomic status; educational level; number of children.
			7	Risk factors for CC: - age of starting sexual intercourse; number of intercourse partners; previous VPH vaccine
				·
Data sources measurement	8*	For each variable of interest, give sources of data and details of methods of assessment (measurement). Describe comparability of assessment methods if there is more than one group	8,6,7	The sources of data were the answers of the patient and the results of laboratory.
Bias	9	Describe any efforts to address potential sources of bias	6	Bias of information due to Spanish language comprehension was controlled by intervention of women
Study size	10	Explain how the study size was arrived at	5	The study size depended of the number of women who attended the screening campaigns

Quantitative variables	11	Explain how quantitative variables were handled in the analyses. If applicable, describe whichgroupings were chosen and why	9	The statistical analyses were mainly descriptive. For univariate analysis, quantitative variables were analyzed using medians andranges. Qualitative variables werereported using percentages and frequency tables.<br>-Age was grouped by five year-periods.<br>-Number of sexual partners was grouped as: 1; 2 or 3; 4 or 5; 6 ormore.<br>-Number of children was groupedas 3 or less; and more than 3.
Statistical methods	12	(*a*) Describe all statistical methods, including those used to control for confounding	9	Means, medians, Odds Ratio and Confidence Intervals 95%; exact Fischer and Chi square test.
		(*b*) Describe any methods used to examine subgroups and interactions	9, 10	.Relation between risk factors and HPV-HR was explored by Fisher's test considering a possible association at a significance level of 0.05.
		(*c*) Explain how missing data were addressed	23	Missing data were accounted for in the denominators of each proportion.
		(*d*) *Cohort study*—If applicable, explain how loss to follow-up was addressed<br>*Case-control study*—If applicable, explain how matching of cases and controls was addressed<br>*Cross-sectional study*—If applicable, describe analytical methods taking account of samplingstrategy	5	There was no sampling process. All patients who assisted for the screening test and accomplished inclusion criteria were included.
		(*e*) Describe any sensitivity analyses		
**Results**				

Participants	13*	(a) Report numbers of individuals at each stage of study—eg numbers potentially eligible, examined for eligibility, confirmed eligible, included in the study, completing follow-up, andanalysed	6<br>10	304 women who attended the screening campaign were included.<br>Of these, 24 were excluded from<br>the analysis for this study because<br>they belonged to ethnic groups<br>other than indigenous or did not belong to any.<br>The total of women included in thestudy was 280, belonging to seven indigenous ethnicities
		(b) Give reasons for non-participation at each stage		
		(c) Consider use of a flow diagram		
Descriptive data	14*	(a) Give characteristics of study participants (eg demographic, clinical, social) and information on exposures and potential confounders	10	Table N. 1
		(b) Indicate number of participants with missing data for each variable of interest	23	There was 20% (56/280) of missing data in the variable “Number of sexual partners.”
		(c) *Cohort study*—Summarise follow-up time (eg, average and total amount)		
Outcome data	15*	*Cohort study*—Report numbers of outcome events or summary measures over time<br>*Case-control study*—Report numbers in each exposure category, or summary measures of exposure		

		Cross-sectional study—Report numbers of outcome events or summary measures	10- 16	-Table 1. Sociodemographic characteristics and Risk factors in women with and withut HPV infection<br>-Table 2. Results of liquid-based cytology of cervical samples from HPV positive women from an Indigenous reserve in Colombia<br>Table 3 Single or multiple infection in women HPV positive<br>Table 4 Distribution of HR-HPV genotypes.<br>Table 5. HR-HPV genotypes combination.<br>Fig 1. Distribution of Global and HR-HPV infections by age<br>-Fig. 2. Frequency of HR-HPV genotypes<br>-Fig. 3. Distribution of HR-HPV according to single or coinfection presentation<br>-Fig. 4. Distribution of LR-HPV according to single or coinfection presentation
Main results	16	(*a*) Give unadjusted estimates and, if applicable, confounder-adjusted estimates and their precision (eg, 95% confidence interval). Make clear which confounders were adjusted for andwhy they were included<br>(*b*) Report category boundaries when continuous variables were categorized<br>(*c*) If relevant, consider translating estimates of relative risk into absolute risk for a meaningful time period		
Other analyses	17	Report other analyses done—eg analyses of subgroups and interactions, and sensitivity analyses		
**Discussion**				

Key results	18	Summarise key results with reference to study objectives	2	Abstract
Limitations	19	Discuss limitations of the study, taking into account sources of potential bias or imprecision. Discuss both direction and magnitude of any potential bias	23<br><br>8	Discussion: Limitations: sample size; sample representativeness and missing dates<br>Possible language comprehension bias was controlled with translation in ancestral languages.
Interpretation	20	Give a cautious overall interpretation of results considering objectives, limitations, multiplicity of analyses, results from similar studies, and other relevant evidence	18<br><br>8<br><br>21, 23	-Cautious related to comparison of prevalence when different techniques of DNA-HPV are used.<br>-Cautious related to inference because the size and not representativeness of sample<br>- Cautious related to obtain conclusions based on race or habits of Indigenous people.
Generalisability	21	Discuss the generalisability (external validity) of the study results	18, 21,23	
**Other information**				
Funding	22	Give the source of funding and the role of the funders for the present study and, if applicable, for theoriginal study on which the present article is based		Out of the text of the article, in a special section.<br>Ministerio de Ciencia, Tecnología e Innovación; Fundación Universitaria de Ciencias de la Salud; Universidad El Bosque; and Universidad de Ciencias Aplicadas y Ambientales

*Give information separately for cases and controls in case-control studies and, if applicable, for exposed and unexposed groups in cohort and cross-sectional studies.

Note: An Explanation and Elaboration article discusses each checklist item and gives methodological background and published examples of transparent reporting. The STROBE checklist is best used in conjunction with this article (freely available on the Web sites of PLoS Medicine at http://www.plosmedicine.org/, Annals of Internal Medicine at http://www.annals.org/, and Epidemiology at http://www.epidem.com/). Information on the STROBE Initiative is available at www.strobe-statement.org.
